# Forme très amyotrophique et invalidante d'une sclérodermie masculine

**DOI:** 10.11604/pamj.2015.22.99.7212

**Published:** 2015-10-05

**Authors:** Olfa Berriche, Zohra Elati

**Affiliations:** 1 Service de Médecine Interne, Hopital Taher Sfar, Mahdia, Tunisie

**Keywords:** Sclérodermie systémique, ésions de sclérose cutanée, physiopathologie, systemic scleroderma, lesions of cutaneous sclerosis, physiopathology

## Image in medicine

La sclérodermie systémique (SS) est une maladie rare qui se caractérise par des anomalies de la microcirculation et des lésions desclérose cutanée et/ou viscérale. Les lésions de sclérose cutanée peuvent être modestes ou étendues. Elle touche avec prédilection les femmes, sa physiopathologie est mal connue et chez l'homme l'existence d'un facteur déclenchant environnemental est discutée. Le sexe masculin semble être, un critère péjoratif d’évolution. A ce propos, nous rapportons un cas exceptionnel de SS masculine à présentation atypique. Un malade, âgé de 41 ans, suivi pour uneSS. Le diagnostic était retenu devant une sclérose cutanée diffuse et une sclérodactylie, il avait par ailleurs une raréfaction des anses capillaires à la capillaroscopie et une hypotonie du sphincter du bas œsophage à la manométrie. L'interrogatoire ne retrouvait pas de facteur déclenchant. L'examen physique trouvait une sclérose diffuse avec amyotrophie très importante et limitation des mouvements au niveau des 4 membres à l'origine d'une impotence fonctionnelle. Le bilan d'amaigrissement (bilan thyroïdien, glycémie, enzymes musculaires, marqueurs tumoraux…) était normal. La biopsie cutanée était en faveur d'une SS. IL était traitée par de la colchicine et des séances régulières de rééducation physique, l’évolution est restée stable.

**Figure 1:**
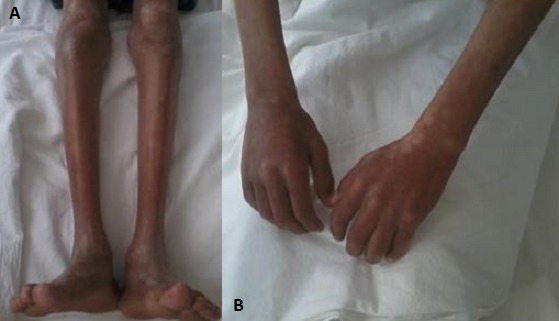
(A) sclérose cutanée au niveau des 2 membres inférieurs avec amyotrophie importante; (B) sclérose cutanée au niveau des 2 membres supérieurs avec amyotrophie importante et troubles de la pigmentation

